# Application of lipid nanovesicle drug delivery system in cancer immunotherapy

**DOI:** 10.1186/s12951-022-01429-2

**Published:** 2022-05-06

**Authors:** Yinan Ding, Luhong Wang, Han Li, Fengqin Miao, Zhiyuan Zhang, Chunmei Hu, Weiping Yu, Qiusha Tang, Guoliang Shao

**Affiliations:** 1 Medical School of Southeast University, Nanjing, 210009 China; 2 Department of Tuberculosis, the Second Affiliated Hospital of Southeast University (the Second Hospital of Nanjing), Nanjing, 210009 China; 3 Department of Neurosurgery, Nanjing Jinling Hospital, Nanjing University, Nanjing, 210002 China; 4 Department of Interventional Oncology, The Cancer Hospital of the University of Chinese Academy of Sciences (Zhejiang Cancer Hospital), Institute of Basic Medicine and Cancer (IBMC), Chinese Academy of Sciences, Hangzhou, 310022 Zhejiang China

**Keywords:** Cancer immunotherapy, Nanovesicles, Liposomes, Cell membrane vesicles, Bacterial outer membrane vesicles, Extracellular vesicles, Hybrid nanovesicles

## Abstract

Immunotherapy has gradually emerged as the most promising anticancer therapy. In addition to conventional anti-PD-1/PD-L1 therapy, anti-CTLA-4 therapy, CAR-T therapy, etc., immunotherapy can also be induced by stimulating the maturation of immune cells or inhibiting negative immune cells, regulating the tumor immune microenvironment and cancer vaccines. Lipid nanovesicle drug delivery system includes liposomes, cell membrane vesicles, bacterial outer membrane vesicles, extracellular vesicles and hybrid vesicles. Lipid nanovesicles can be used as functional vesicles for cancer immunotherapy, and can also be used as drug carriers to deliver immunotherapy drugs to the tumor site for cancer immunotherapy. Here, we review recent advances in five kinds of lipid nanovesicles in cancer immunotherapy and assess the clinical application prospects of various lipid nanovesicles, hoping to provide valuable information for clinical translation in the future.

## Background

As one of the diseases with the highest mortality, a cancer diagnosis is usually considered a death sentence for most people. To date, thousands of scientists and doctors have spent great deal of energy in cancer research, and their efforts have greatly improved the survival chances of cancer patients. According to authoritative statistics, the mortality rate of cancer has decreased since 1991 and has continued to decline through 2017, which is mainly due to the declining mortality of breast cancer, colorectal cancer, lung cancer and prostate cancer [1–4].

Currently, a variety of cancer therapies have been applied in the clinic, such as surgery, radiotherapy, chemotherapy, immunotherapy, gene therapy, radionuclide therapy, endocrine therapy and photodynamic therapy (PDT) [5–10]. However, although there are a large number of cancer-related basic research studies, drug developments and clinical trials every year, the limitations of antitumor drugs are consistently an unavoidable topic, especially the side effects [11–16]. Most of the side effects of anticancer drugs are due to toxicity to nontumor tissues, especially for traditional therapies. For example, chemotherapy can cause the cardiotoxicity, peripheral neurotoxicity, myelosuppression, alopecia, nausea and vomiting [17–21]. To maximize clinical efficacy while minimizing side effects, cancer-targeted nanoparticles have been tried to be used in cancer therapy. Due to the unique nanoscale size of nanoparticles and the special tumor pathological environment, nanoparticles can easily enter and be retained in tumor tissue, which is also known as the enhanced permeability and retention (EPR) effect [22–24]. For example, Liu et al. designed a highly effective supramolecular nanomicellar drug formulation carrying doxorubicin, which exhibited potent anticancer activity for pancreatic ductal adenocarcinoma (PDAC) [25]**.** Moreover, to further improve the aggregation ability of nanoparticles in tumor tissues, most scientists modify the surface of nanoparticles to actively target tumors, to improve their targeting ability. For instance, He et al. designed Angiopep-2 coupled polymersomes targeting glioblastoma to increase the concentration of drugs in glioblastoma and reduce the aggregation of drugs in noncancer areas, which achieved a great curative effect [26].

According to the Web of Science, there are thousands of studies about the application of nanoparticles in cancer therapy annually. However, only a few nanoparticles have been applied in the clinic. Most studies remain at the stage of animal experiments. Here we list three main reasons that hinder the clinical translation of nanoparticles. Firstly, most nanoparticles have complex components, which make them difficult to metabolize. Secondly, the high cost of production and difficult quality control would greatly increase the medical expenses of patients, which limits the popularization and application of nanoparticles in the clinic. Lastly, unquantifiable toxicology and pharmacokinetics lead to uncertain safety effects and therapeutic effects [27]. Nevertheless, in the past few decades, a number of nanoparticles have been approved for cancer therapy by the Food and Drug Administration (FDA), most of which are liposome-based nanoparticles [28], such as Eligard [29], DaunoXome [30], Marqibo [31], Onivyde [32], Doxil [33], Abraxane [34], Ontak [35] and Nanotherm [36] (Table 1). Similar to liposomes, cell membrane vesicles, bacterial outer membrane vesicles (OMVs) and extracellular vesicles (EVs) also have phospholipid membranes, which possess good biocompatibility and efficient drug loading capacity and exhibit potential for clinical translation. Here, we classified liposomes, cell membrane vesicles, bacterial outer membrane vesicles, extracellular vesicles and hybrid vesicles as lipid nanovesicles.

**Table 1:** List of FDA-Approved Nanomedicines for Cancer Therapy

Name	Material Description	Nanoparticle Advantage	Indication(s)	Year(s) approved
Eligard^®^ (Tolmar)	Leuprolide acetate and polymer (PLGH (poly (DL-Lactide-coglycolide))	Controlled delivery of payload with longer circulation time	Prostate Cancer	2002
DaunoXome^®^ (Galen)	Liposomal Daunorubicin	Increased delivery to tumor site; lower systemic toxicity arising from side-effects	Kaposi’s Sarcoma	1996
Marqibo^®^ (Onco TCS)	Liposomal Vincristine	Increased delivery to tumor site; lower systemic toxicity arising from side-effects	Acute Lymphoblastic Leukemia	2012
Onivyde^®^ (Merrimack)	Liposomal Irinotecan	Increased delivery to tumor site; lower systemic toxicity arising from side-effects	Pancreatic Cancer	2015
Doxil^®^/Caelyx™ (Janssen)	Liposomal doxorubicin	Improved delivery to site of disease; decrease in systemic toxicity of free drug	Kaposi’s Sarcoma; Ovarian cancer; multiple myeloma	1995 2005 2008
Abraxane^®^/ABI-007 (Celgene)	Albumin-bound paclitaxel nanoparticles	Improved solubility; improved delivery to tumor	Breast cancer; NSCLC; Pancreatic cancer	2005 2012 2013
Ontak^®^ (Eisai Inc)	Engineered Protein combining IL-2 and diphtheria toxin	Targeted T-cell specificity; lysosomal escape	Cutaneous T-Cell Lymphoma	1999
Nanotherm^®^ (MagForce)	Iron oxide		Glioblastoma	2010

Reprinted with permission from ref. [28]. Copyright (2016) Springer Nature

NSCLC non-small cell lung cancer

**Fig. 1:**
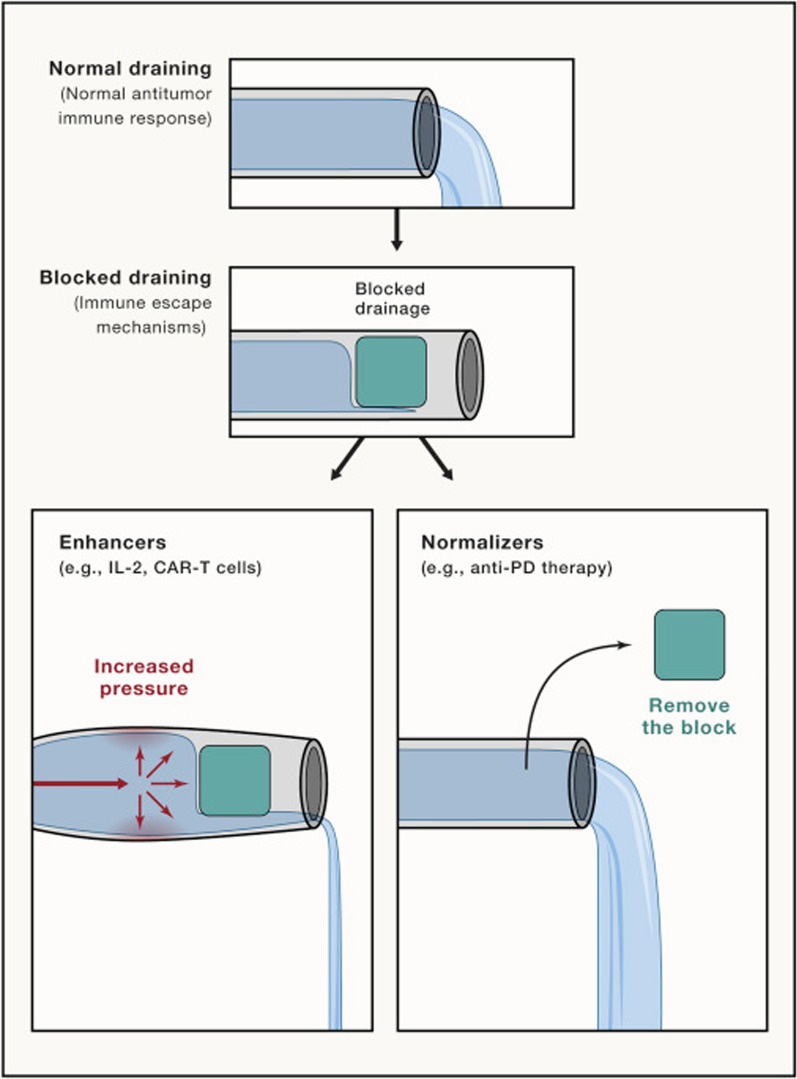
Schematic illustration of the Immune-Normalization versus Immune-Enhancement Approaches. Using proper flow and drainage of a pipeline as a comparison for the antitumor immune response. The flow of the pipeline can be insufficient when a blockade impairs flow, as the antitumor immune response can be insufficient when there is an immune impairment. The immune enhancement approach is illustrated as an increase inflow or pressure to return to proper function/flow with the risk of breaking the pipe (adverse effects). In contrast, the immune normalization approach would be to identify and try to unblock this specific blockage and restore the flow. Reprint with permission from [47]. Copyright 2018, Elsevier

In 2018, Chen and his colleagues proposed that the concept of tumor immunotherapy should change from enhancement to normalization. They concluded that traditional tumor immunotherapy mainly focuses on enhancing immunity by using effector cells/molecules to stimulate the immune system to directly attack tumor cells, which is also called “passive” immunotherapy, such as antibody-targeted therapies (e.g., Her2/neu monoclonal antibody (mAb) [37], anti-EGFR mAb [38] and anti-CD20 mAb) and adoptive immune cell therapies (e.g., macrophage-based adoptive cell therapy [39], chimeric antigen receptor (CAR)-T [40], CAR-NK [41] and adoptive CD8^+^ T-cell therapy [42]). Recently, immune checkpoint related therapies have attracted more attention, including programmed death-1 (PD-1), programmed death ligand 1 (PD-L1) [43, 44] and CD47 [45, 46]. Moreover, the FDA has approved several anti-PD-1/PD-L1 drugs since 2014. In addition, there are a variety of immunomodulatory drugs and cancer vaccines that can stimulate the immune system modulating the cancer immune microenvironment and improving the local immune status of cancer, and they are defined as “active” immunotherapies. In summary, they presented a theory of water flow in pipelines (Fig. 1), and an unobstructed pipeline represents normal immunity. Under pathological conditions, the pipeline is blocked, which means that the immune response is insufficient. The immune enhancement approach can slightly improve the flow by increasing the pressure with the risk of breaking the pipeline, which means that improving the immune response by enhancers would also cause adverse effects. In contrast, the immune normalization approach identifies and removes the block and restores the flow without pipeline damage [47].

**Fig. 2:**
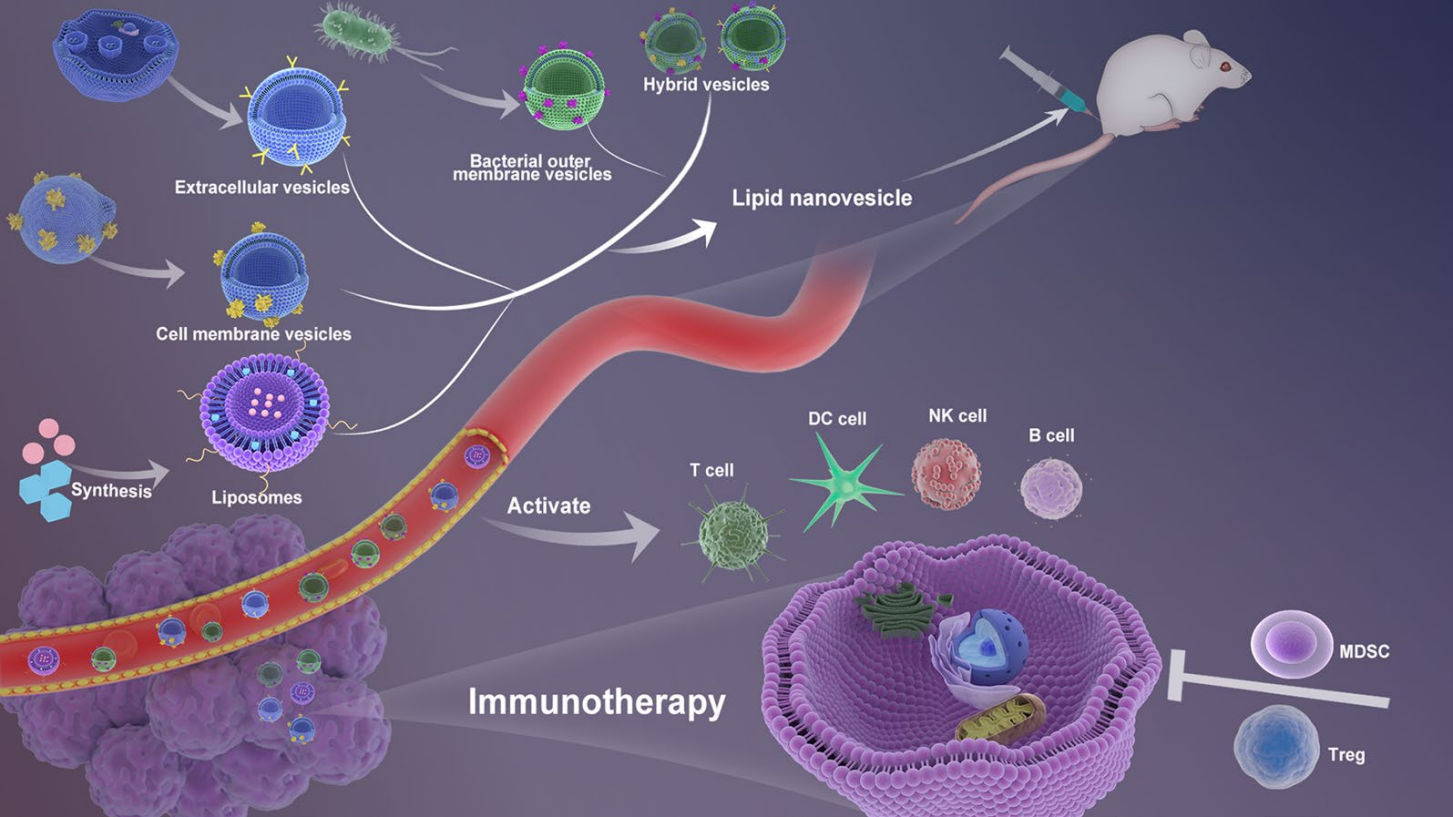
The application of lipid nanovesicle drug delivery system in cancer immunotherapy

Here, we focus on the recent advances in lipid nanovesicle drug delivery systems (LNDDSs) in cancer immunotherapy and from these cutting-edge studies, we analyze the current challenges and future perspectives of LNDDS for translational in cancer immunotherapy (Fig. 2).

### Liposomes in cancer immunotherapy

Liposomes were first discovered in 1965 by Bangham et al. [48]. Liposomes are spherical lipid bilayer vesicles with hydrophobic and hydrophilic regions. As one of the best candidates for drug delivery systems, liposomes possess good drug loading capacity and biocompatibility. With the development of tumor immunotherapy, how to combine liposome drug delivery systems with immunotherapy has become a new research topic [49, 50].

As one of the most successful types of immunotherapy, immune checkpoints, as potential cancer immunotherapy targets, have also been applied to liposome drug delivery systems. Considering that cancer cells can escape immune surveillance by surface overexpression of CD47 and PD-L1, Shu et al. designed an epithelial cell adhesion molecule (EpCAM)-targeted cationic liposome with si-PD-L1 and si-CD47, which could actively target EpCAM overexpressing cancer cells and knockdown the PD-L1 and CD47 proteins. This liposome-based dual-blockade cancer cell immune checkpoint therapeutic strategy effectively activated anticancer T cells and nature killer (NK) cells, and promoted the release of cytokines such as interferon-γ (IFN-γ) and interleukin-6 (IL-6), which exhibit good anticancer abilities [51].

**Fig. 3:**
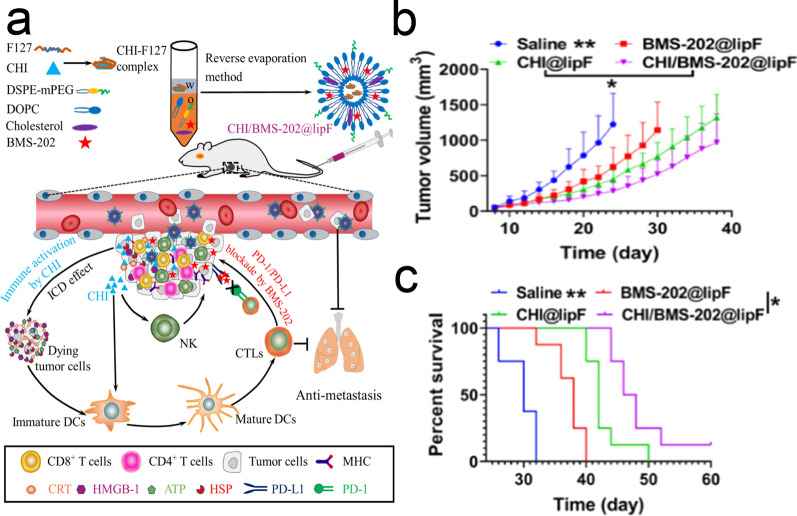
CHI/BMS-202@lipF-Mediated Synergistic TNBC Treatment. **a** Schematic Illustration of CHI/BMS-202@lipF-Mediated Synergistic TNBC Treatment. **b** Treatment schedule for CHI/BMS-202@lipF-mediated antitumor combination therapy in vivo. **c** Average tumor volume. **d** Survival rate of mice with various treatments (n = 8). Reprint with permission from [54]. Copyright 2021, American Chemical Society

In the clinic, due to individual differences and cancer heterogeneity, traditional anti-PD-1/PD-L1 therapy cannot achieve the ideal therapeutic effect for most patients. To solve this problem, scientists have combined anti-PD-1/PD-L1 therapy with other therapeutic strategies, such as Improving cancer immunogenicity and activating anti-cancer immune cells (dendritic cells (DCs), T cells, NK cells, tumor-associated macrophages (TAMs), etc.) and decreasing the number of suppressor immune cells (e.g., T regulatory cells (Tregs) and myeloid-derived suppressor cells (MDSCs)). Chen et al. prepared a liposome that dual-modified anti-PD-L1 and mannose for targeting PD-L1 on cancer cells and mannose receptor (MR, aka CD206) on TAMs. Moreover, this liposome was encapsulated with an antiangiogenic drug (regorafenib) and an mTOR inhibitor (rapamycin) to ameliorate the tumor immune microenvironment (TIME). This kind of liposome delivery system can simultaneously inhibit angiogenesis, repolarize TAMs, inhibit glycolysis, reprogram immune cells and effectively reduce the tumor volume and is a promising liposome delivery system for cancer combination therapy [52]. Guido Kroemer and Laurence Zitvogel discovered and put forward the concept of immunogenic cell death (ICD). ICD can improve the immunogenicity of tumors and turn “cold tumors” into “hot tumors”, so that immune cells can recognize tumors easily, which provides a new idea for cancer treatment [53]. Tu et al. use liposome as the carrier, co-deliver the chidamide (CHI), an epigenetic modulator, and BMS-202 (a PD-L1 inhibitor) as a synergistic cancer treatment strategy. They verified that CHI could induce ICD in triple-negative breast cancer (TNBC) and enhance cancer immunogenicity. In addition, CHI can increase the levels of major histocompatibility complex-I (MHC-I) and MHC-II on TNBC cells, which can promote antigen presentation and T-cell recognition. Furthermore, CHI can promote DC maturation and activate NK cells. Combined with anti-PD-L1 drugs, this liposome can effectively inhibit tumor growth and metastasis [54] (Fig. 3). Xiong et al. designed a two-in-one nanoplatform (IR775@Met@Lip), and the photosensitizer IR775 and metformin were encapsulated in liposomes. PDT, as a promising strategy, can generate reactive oxygen species (ROS) to damage cancer cells and promote anticancer immunity by increasing the secretion of IFN-γ. However, the increased secretion of IFN-γ induced by PDT would extremely increase the expression of PD-L1 on the cancer cells, and it would weaken the function of T cells. Metformin (Met), as an oral hypoglycemic drug, is used as a treatment drug for type II diabetes clinically [55, 56]. Recent studies have found that metformin can reduce the expression of PD-L1 on the surface of cancer cells [57]. In this liposome drug delivery system, Met can remedy the side effects of PDT and reduce the expression of PD-L1 on the tumor surface. Combined with the advantages of PDT in tumor treatment, this IR775@Met@Lip system can be a promising cancer therapy modality [58].

MDSCs, as inhibitory cells, exist in the tumor environment. The immune response can be obstructed by MDSCs through various mechanisms [59]. As one of the star products in liposomes, Doxil has been widely used in the clinic. Compared with free doxorubicin (DOX), Doxil exhibits lower cardiotoxicity [60, 61]. Recently, DOX has been proven to have the potential to enhance immunity and inhibit the population and function of MDSCs [62]. Jamshid et al. designed a new liposomal platform, modified P5 peptide on Doxil. This liposome can stimulate the immune system and decrease the effect of MDSCs, which can effectively inhibit tumor growth [63].

A recent study showed that indoleamine 2,3-dioxygenase 1 (IDO-1) is highly expressed in tumors and can promote the induction of Tregs and inhibit the growth of infiltrating T cells [64]. Several IDO-1 inhibitors have shown an efficient immunomodulatory ability by reversing the immunosuppressive TIME [65–67]. Nevertheless, limited by the water solubility and bioavailability of IDO inhibitors, liposomes are a suitable drug carrier for IDO inhibitors [68, 69]. To solve the problem of drug delivery of IDO inhibitors, Mei et al. encapsulated the indoximod (IND) prodrug in the lipid bilayer of liposomes combined with mitoxantrone (MTO) in the hydrophilic layer. Compared with liposomal MTO, the immune response was significantly enhanced by co-delivery an IDO-1 inhibitor in IDO-overexpressed cancers, such as renal cancer (RENCA) and breast cancer (4T1 and EMT6) [67].

**Fig. 4:**
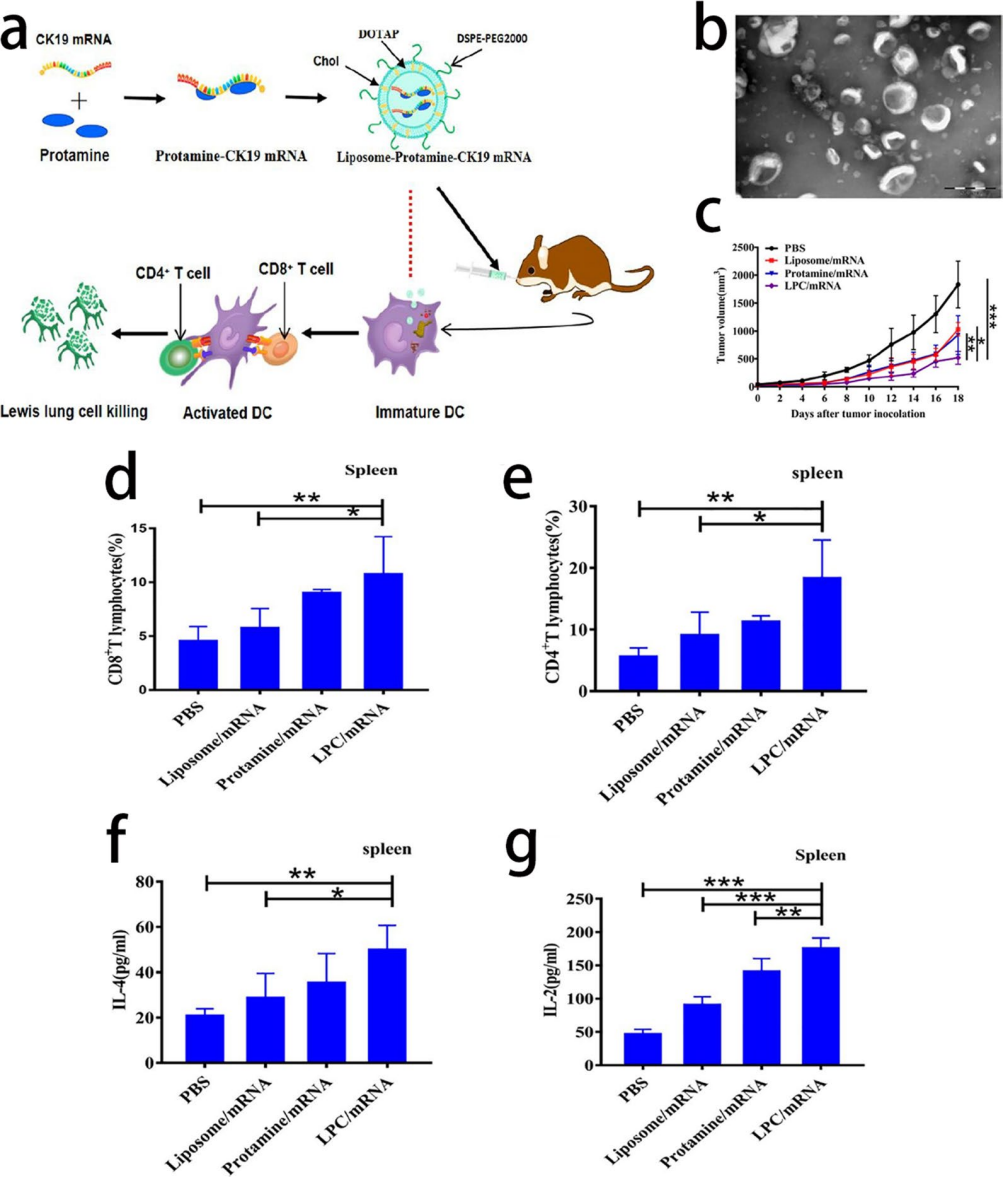
**a** Schematic illustration of synthesis process of cationic liposome/protamine complex (LPC) and immunotherapy in Lewis lung cell. **b** Transmission electron micrograph of LPC/mRNA. Scale bar = 200 nm. **c** Tumor growth of mice bearing LLC during treatment. Stimulation of CD4^+^T cells (**d**) and CD8^+^T cells **e** in the spleen. Data are presented as the mean ± SD (n = 4). *p < 0.05, ***p < 0.001. **f** and **g** Spleen cells from mice after administration of different formulations collected 1 week after the final immunization and cultured with CK19 for 72 h. The supernatants were collected and the production of cytokines IL-4 and IL-2 was measured. The results are presented as the mean ± SD of experiments performed in triplicate. Data are presented as the mean ± SD *p < 0.05, **p < 0.01, ***p < 0.001. Reprint with permission from [74]. Copyright 2020, Elsevier Inc

Since the end of 2019, the world has been experiencing a severe pandemic due to the outbreak of coronavirus disease-19 (COVID-19) [70, 71]. To date, the most effective preventive measure is vaccination, and among all of the vaccines, the messenger ribonucleic acid (mRNA) vaccine is the most effective and widely used vaccine. Most FDA-approved COVID-19 vaccines are delivered by lipid nanoparticles, such as BNT162b2 (BioNTech/Pfizer) and mRNA-1273 (Moderna) [72]. Similarly, although there are officially approved products in the clinic, mRNA vaccines still exhibit great potential in cancer immunotherapy [73]. As one of the most popular mRNA delivery carriers, cationic liposomes can concentrate mRNA and can be easily absorbed by antigen-presenting cells (APCs). Mai et al. attempted to encapsulate positively charged protamine concentrated mRNA with cationic liposomes. The nasal administration of the cationic liposome/protamine complex can promote the maturation of dendritic cells and contribute to inducing an antitumor immune response in vivo, to inhibit the growth of tumors, which proves that cationic liposomes can be used as an efficient and safe carrier for mRNA cancer vaccine in future clinical translation [74] (Fig. 4).

In summary, as one of the best nano drug carriers, liposomes are convenient to prepare and possess high drug encapsulation efficiency, which exhibit great clinical translation potential. However, liposomes don’t have therapeutic function. In addition to conventional drug loading, we also need to endow them with some functions through chemical or physical methods, such as targeting ability, immunotherapeutic ability and so on. However, the preparation process of large-scale functional liposomes is complex and cumbersome, which makes the preparation process difficult to quality control and increases the difficulty of clinical translation.

### Cell membrane vesicles in cancer immunotherapy

After the great translational success of liposomes in the clinic, biomembrane-based vesicles have also been considered to be an efficient drug delivery system [75]. Recently, a variety of vesicles derived from different cell membranes have been developed, such as vesicles derived from the membrane of leukocytes, red blood cells, platelets, mesenchymal stem cells and cancer cells etc. [76, 77].

**Fig. 5:**
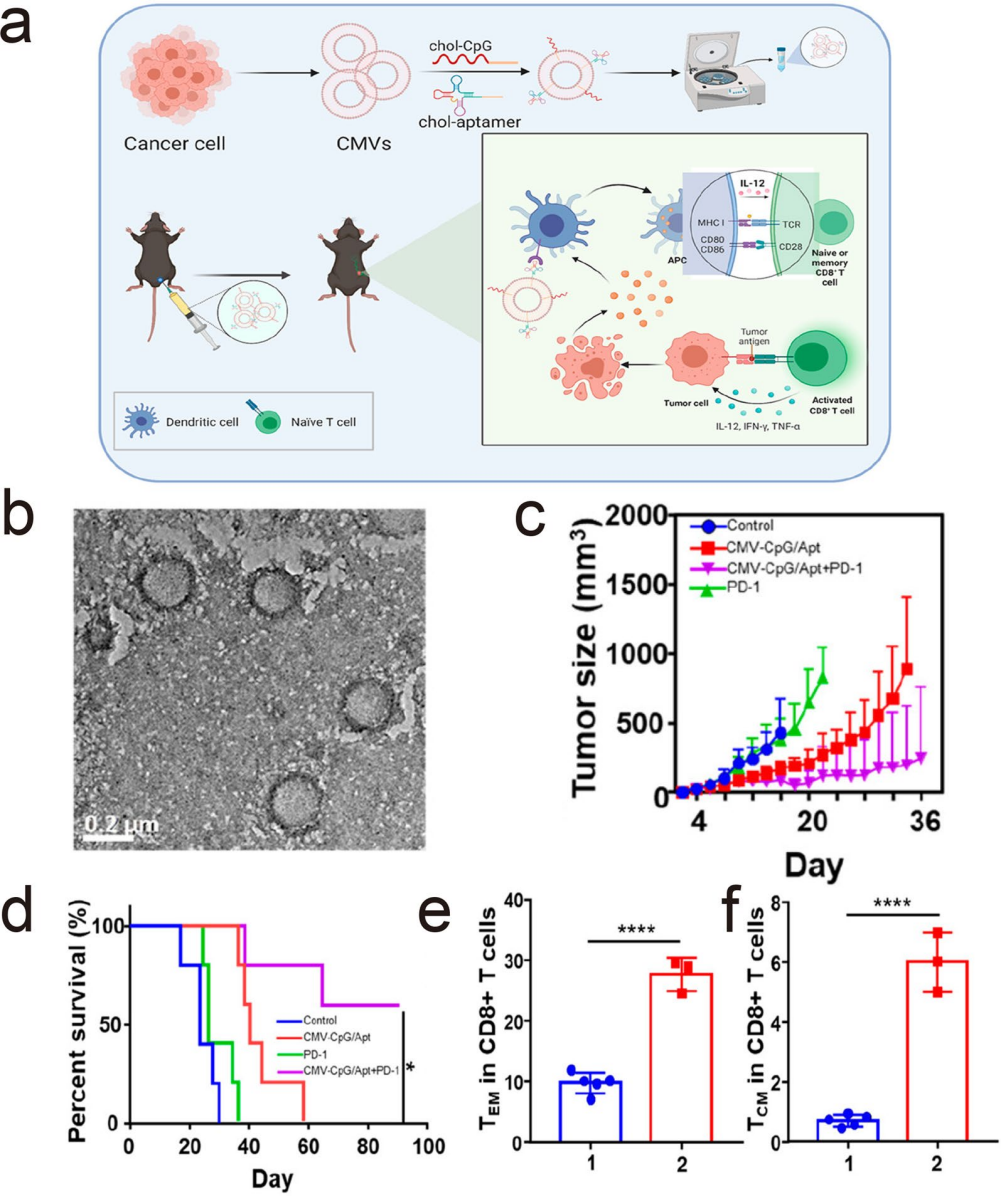
**a** Schematic illustration to show the preparation of nano vaccines from tumor-cell-derived CMVs and their functions to induce antitumor immunity. **b** A TEM image of cancer CMVs. **c** The survival curves of different groups of mice with CT26 tumors after various treatments. **d** Growth curves of CT26 tumors on mice after various treatments. The statistical data of effective memory T cells (T_EM_) **e** and central memory T cells (T_CM_) (**f**) in the peripheral blood before rechallenging the mice with secondary tumors. Reprint with permission from [83]. Copyright 2021, America Chemical Society

**Fig. 6:**
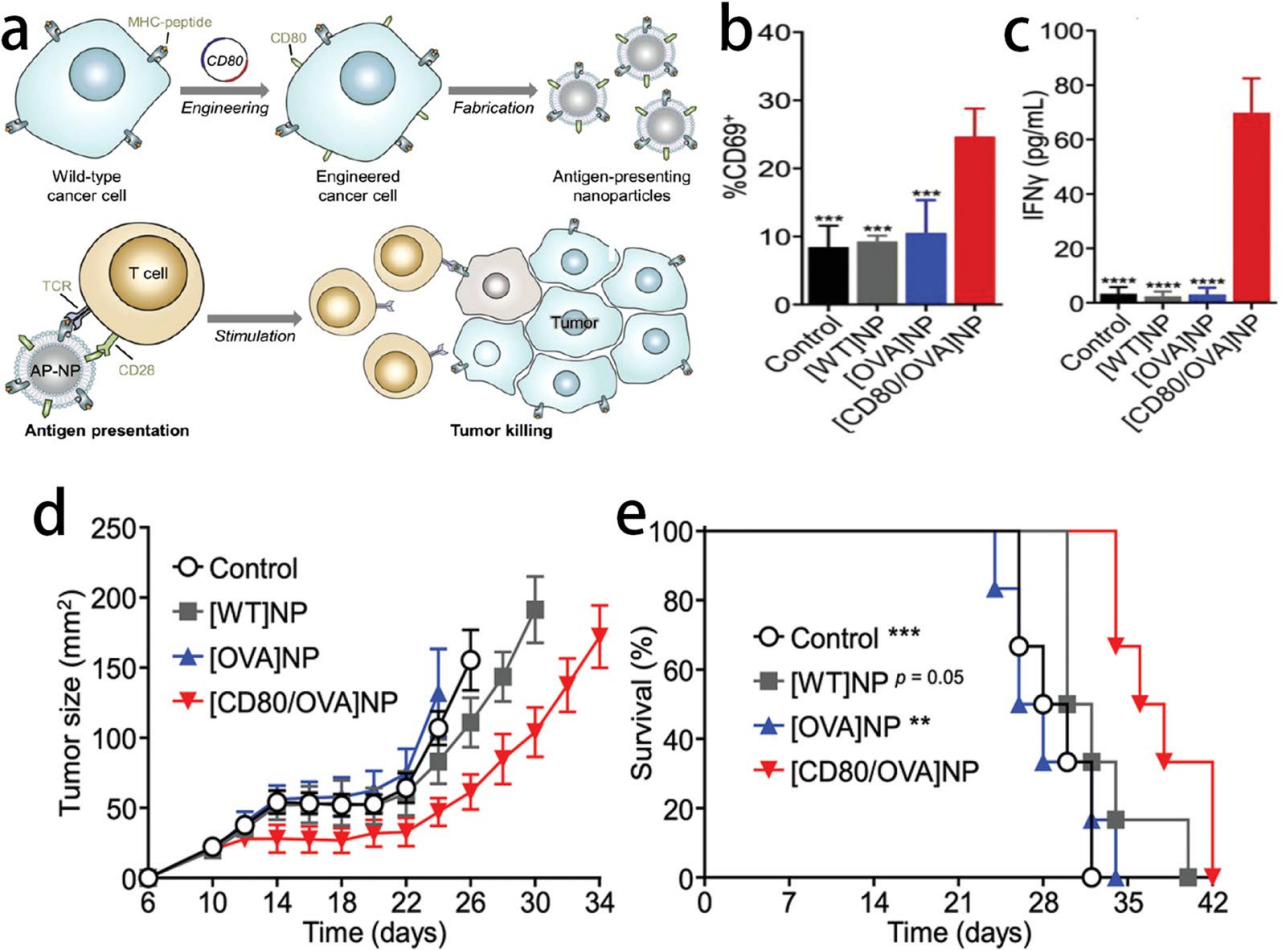
**a** Schematic of engineered cell-membrane-coated nanoparticles for direct antigen presentation. **b** Expression of CD69 by OT-I CD8 + T cells in the draining lymph nodes 3 days after administration of [CD80/OVA]NPs or control nanoparticles into C57BL/6 mice adoptively transferred with OT-I splenocytes (n = 4, mean + SD). **c** Secretion of IFN-γ by draining lymph node cells 4 days after administration of [CD80/OVA]NPs or control nanoparticles into C57BL/6 mice adoptively transferred with OT-I splenocytes (n = 3, mean + SD). Average tumor sizes (**f**) and survival (**h**) over time for the therapeutic efficacy study (n = 6; mean ± SEM). ***p < 0.001, ****p < 0.0001 (compared to [CD80/OVA]NP); one-way ANOVA. Reprint with permission from [86]. Copyright 2020, WILEY‐VCH Verlag GmbH & Co. KGaA, Weinheim

Among all kinds of cell sources, cancer cells can be successfully applied in cancer immunotherapy, especially as vaccine carriers [78–81]. Christopher et al. prepared a tumor membrane vesicles (TMVs) vaccine, isolated TMVs from 4T1 tumor tissue, and modified immunostimulatory IL-12 and B7-1 (CD80) molecules on the surface of TMVs. TMV-based vaccine-mediated immunotherapy combined with anti-cytotoxic T-lymphocyte-associated protein 4 monoclonal antibody (anti-CTLA-4 mAb) treatment effectively stimulated the immune system, enhanced the immunity of CD8^+^ T cells, reduced tumor metastasis and improved the survival rate [82]. Liu et al. designed a DC targeted nanovaccine by modifying functionalized DC targeted deoxyribonucleic acid (DNA) in TMVs, which combined with immune checkpoint blockade treatment can target DCs and trigger a robust anticancer immune response [83] (Fig. 5). Flavia and his colleagues developed a multistage nanovaccine (NV). They encapsulated thermally oxidized porous silicon (TOPSi) into acetalated dextran (AcDEX) or spermine-modified AcDEX (SpAcDEX) polymeric particles, named TOPSi@AcDEX, which can stimulate DCs and the secretion of proinflammatory cytokines with biodegradability and biocompatibility. Subsequently, TOPSi@AcDEX was encapsulated with TMVs, and the antigenic composition of tumor lysate combined with the adjuvant properties of TOPSi@AcDEX greatly enhanced the anticancer immune system [84]. Recently, Liu et al. designed a tumor vaccine named NP@FM that fused cytomembranes of DCs and tumor cells. Owing to the membrane of DCs, NP@FM emerged antigen-presenting ability. Since NP@FM contained tumor membrane fragments, DCs can recognize NP@FM and induce maturation of DCs, thereby activate antitumor immunity [85]. Furthermore, Jiang et al. engineered cancer cell membrane with co-stimulatory marker, and developed a biomimetic nanoparticle platform that can direct stimulate T cells without professional antigen presenting cells. This novel cancer immunotherapy strategy bypassed the traditional antigen presentation process and exhibited strong immune activation ability [86] (Fig. 6). Meng and his colleagues prepared genetically programmable fusion tumor cell membrane vesicles (Fus-CVs) for double-targeting immune checkpoint blockade therapy, which displayed SIRP-α variants and PD-1. Fus-CVs can significantly increase the phagocytosis of macrophages to cancer cells, promote antigen presentation ability, and activate T-cell immunity. Consequently, Fus-CVs can effectively inhibit the recurrence and metastasis of post-surgery tumor [87]. These results indicated that TMV-based personalized tumor vaccine immunotherapy can effectively improve the immune response and enhance the efficacy of immunotherapeutic drugs, which has great potential for clinical application.

**Fig. 7:**
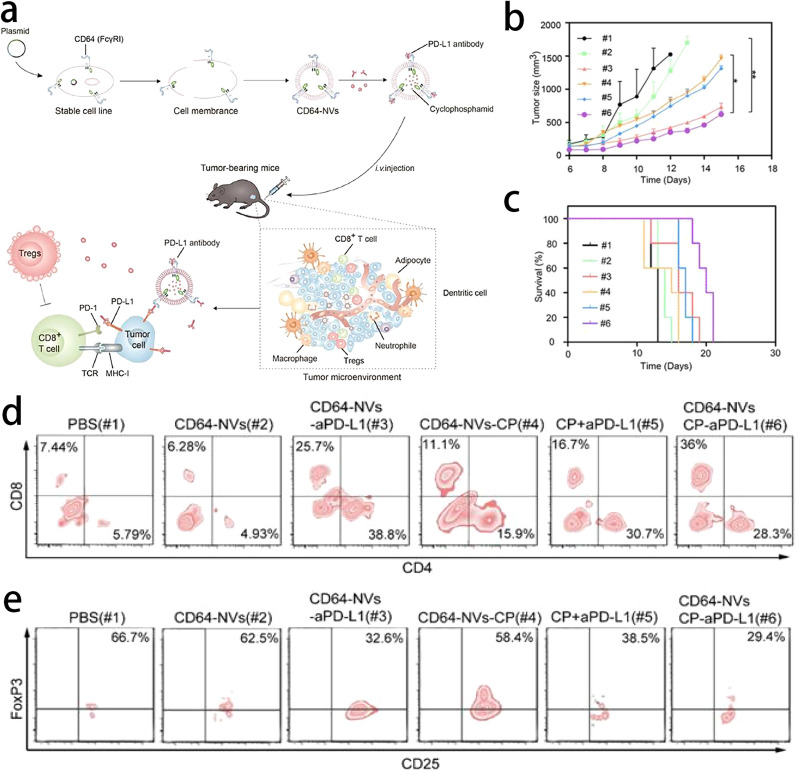
**a** Schematic of preparation of CD64-NVs-aPD-L1-CP and immune boosting mechanism of NVs. **b** Average tumor sizes for the treated mice (n = 5). The experimental data were shown as mean ± SEM. **c** Survival curves for the mice treated with PBS (#1), CD64-NVs (#2), CD64-NVs-aPD-L1 (#3), CD64-NVs-CP (#4), CP + aPD-L1 (#5) and CD64-NVs-CP-aPD-L1 (#6) groups. (n = 5). **d** Representative plots of T cells in tumors of different treatment detected by flow cytometry (Gated on CD3^+^). **e** Representative plots of Foxp3 in Tregs infiltrating in tumors detected by the flow cytometry (gated on CD4^+^). Reprint with permission from [88]. Copyright 2021, Ivyspring International Publisher

**Fig. 8:**
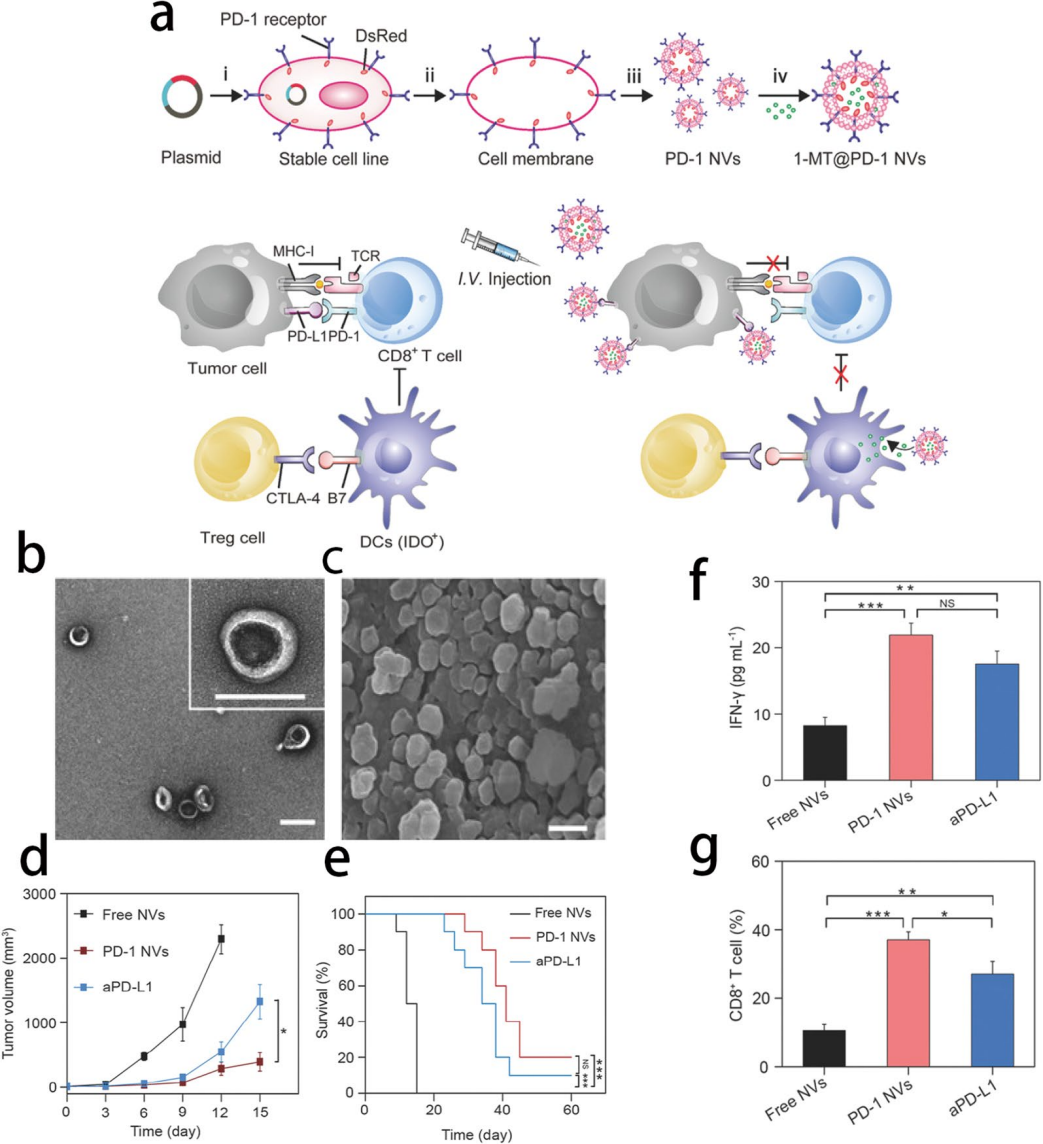
**a** Schematic illustration and characterization of PD-1 blockade cellular NVs for cancer immunotherapy. **b** The TEM image showed the shape and size of PD-1 NVs. Scale bar: 100 nm. **c** Cryoscanning electron microscopy (CSEM) image showed the natural shape of the PD-1 NVs (Scale bar: 100 nm). **d** Average tumor volumes of the treated mice in different groups (n = 7). Error bar, mean ± s.e.m. **e** Survival curves for the mice received the treatment of PD-1 NVs, PD-L1 antibody, and free NVs (n = 10). (f) IFN-γ levels in serum from mice isolated at day 20 after mice received the first indicated treatment (n = 3). Error bar, mean ± s.d. **g** Quantitative analysis of T cells (gated on CD3^+^ cells) in treated tumor analyzed by flow cytometry (n = 3). Error bar, mean ± s.d. Reprint with permission from [89]. Copyright 2018, WILEY‐VCH Verlag GmbH & Co. KGaA, Weinheim

In addition to TMV-based nanoplatforms, genetically engineered cellular vesicles have also demonstrated competitiveness in cancer immunotherapy. Li et al. prepared CD64 presenting cellular NVs derived from overexpressed CD64 HEK 293 T cells so that PD-L1 antibody can easily conjugate with NVs. Additionally, to inhibit Tregs, they encapsulated a low dosage of cyclophosphamide (CP) into NVs. These new NVs enhanced the function of T cells with a PD-L1 antibody and activated CD8 T cells with a low dosage of CP, effectively prolonging the survival time of **mice** [88] (Fig. 7). Similarly, Zhang’s group genetically engineered H293T cells to express PD-1 receptors on the surface of their membranes, and then nanovesicles with PD-1 receptors (PD-1 NVs) were obtained. PD-1 NVs can disrupt the PD-1/PD-L1 immune inhibitory axis. In addition, PD-1 NVs can also carry a variety of different therapeutic drugs to achieve collaborative treatment, which makes PD-1 NVs a multifunctional immunotherapy nanoplatform [89] (Fig. 8).

**Fig. 9:**
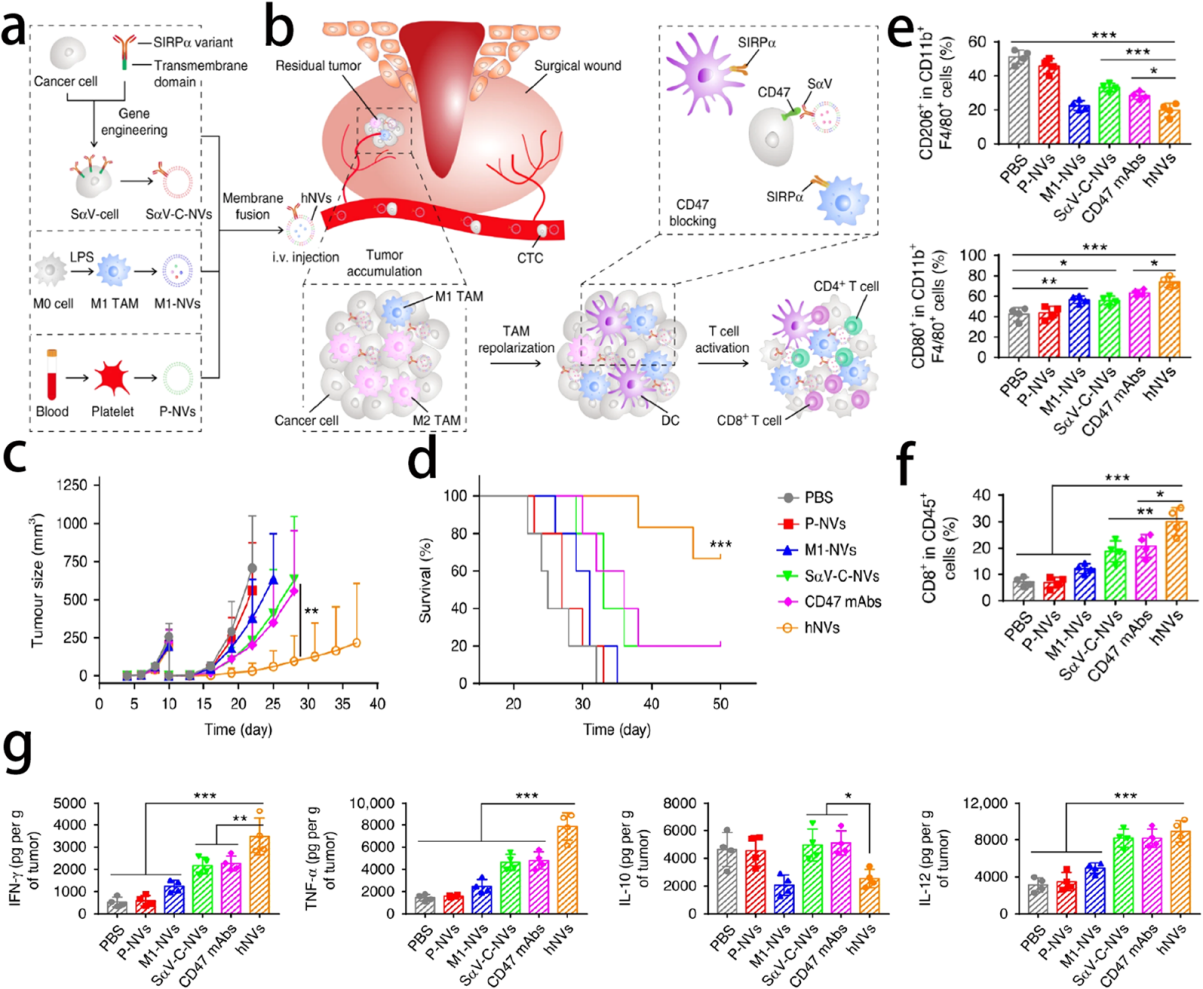
**a** Schematic showing the hNVs consist of engineered SαV-C-NVs, M1-NVs, and P-NVs. **b** Schematic showing the hNVs efficiently interact with CTCs in the blood, accumulate in the post-surgical tumor bed, repolarize TAMs towards M1 phenotype, and block the CD47-SIRPα ‘don’t eat me’ pathway, thus promoting macrophage phagocytosis of cancer cells, as well as boosting antitumor T cell immunity. **c** Average tumor growth kinetics and **d** survival rate in different groups. All data are presented as mean ± S.D. (n = 6 for the hNVs-treated group, n = 5 for the other groups). Statistical significance was calculated via 2way ANOVA with a Tukey’s test d or log-rank (Mantel–Cox) test e. **e** Flow cytometric analysis of M2-like macrophages (CD206^+^) and M1-like macrophages (CD80^+^) in tumor gating on F4/80^+^CD11b^+^CD45^+^ cells. (f) Flow cytometric analysis of CD8^+^ and CD4^+^ T cells in tumor gating on CD45^+^ cells. **g** Cytokine levels in tumors from mice isolated 5 days after different treatments. All data are presented as mean ± S.D. (n = 4). Statistical significance was calculated via ordinary one-way ANOVA with a Tukey’s test. *p < 0.05; **p < 0.01; ***p < 0.001. Reprint with permission from [90]. Copyright © 2020, Springer Nature

As we mentioned before, CD47 is highly expressed on the surface of tumor cells. There are several CD47 antagonists being tested in clinic trails. However, M2-type macrophages restrict the efficacy of CD47 antagonists and CD47 antagonists would cause serious anemia and thrombocytopenia. In order to improve the effect of anti-CD47 immunotherapy, Rao et al. designed a hybrid cell membrane nanovesicles (known as hNVs), which consists of platelet-derived NVs (P-NVs), M1 macrophage-derived NVs (M1-NVs) and cancer cell-derived NV overexpressing high-affinity SIRP-α variants (SαV-C-NVs). The hNVs can accumulate in surgical wound sites and target circulating tumor cells (CTCs) in the blood by P-NVs. M1-NVs can repolarize M2 macrophages into M1 macrophages. In this study, hNVs effectively amplified macrophage responses against cancer recurrence and metastasis after surgery [90] (Fig. 9).

NK cells, as a type of innate immune cell, are the first line of resistance to cancer and infection. What’s more, NK cells can mediate M1-macrophage polarization and specifically target cancer cells by proteins expressed on the surface of NK cell membranes. Based on the characteristics mentioned above, Deng’s group developed a novel immunotherapy strategy based on the NK cell membrane with PDT, which named as NK-NPs. This study demonstrated that NK-NPs can target to cancer cells and enhance M1-macrophage polarization by NK cell membrane to produce anticancer immunity. In addition, PDT can induce ICD to enhance the anticancer immunity efficacy stimulated by NK cell membrane [91].

All in all, cell membrane vesicles are more like a kind of functional liposomes. There are a large number of tumor specific antigens (TSAs) and tumor associated antigens (TAAs) on the surface of tumor derived cell membrane vesicles, which can induce the maturation of APCs, while cell membrane vesicles derived from immune cells can affect the tumor immune microenvironment and play an important role in immune activation effect through their own biological functions. For most cell membrane vesicles, the expected biological functions can be obtained by transfection, so as to activate antitumor immunity. Compared with liposomes, which need to be endowed with biological functions by physical or chemical methods, cell membrane vesicles have inherent advantages in this regard. However, cell membrane vesicles are often obtained from cell lines, which will cause a certain degree of immune rejection. At the same time, high acquisition cost and low yield also limit the clinical translation of cell membrane vesicles.

### Bacterial outer membrane vesicles in cancer immunotherapy

Outer membrane vesicles (OMVs) are spherical nanovesicles that are naturally released by gram-negative bacteria with a lipid bilayer and the size is approximately 20–250 nm [92, 93]. In 1997, Bermudes and his colleagues found that Salmonella can be a novel drug delivery platform for targeting cancer [94]. However, the toxicity of bacteria limits their clinical translation as anticancer carriers in cancer immunotherapy. OMVs are released from bacteria, so that OMVs possess a composition similar to that of bacteria. Additionally, genetically engineered attenuated bacteria can produce OMVs with reduced endotoxicity. Therefore, attenuated OMVs have application value in cancer immunotherapy.

**Fig. 10:**
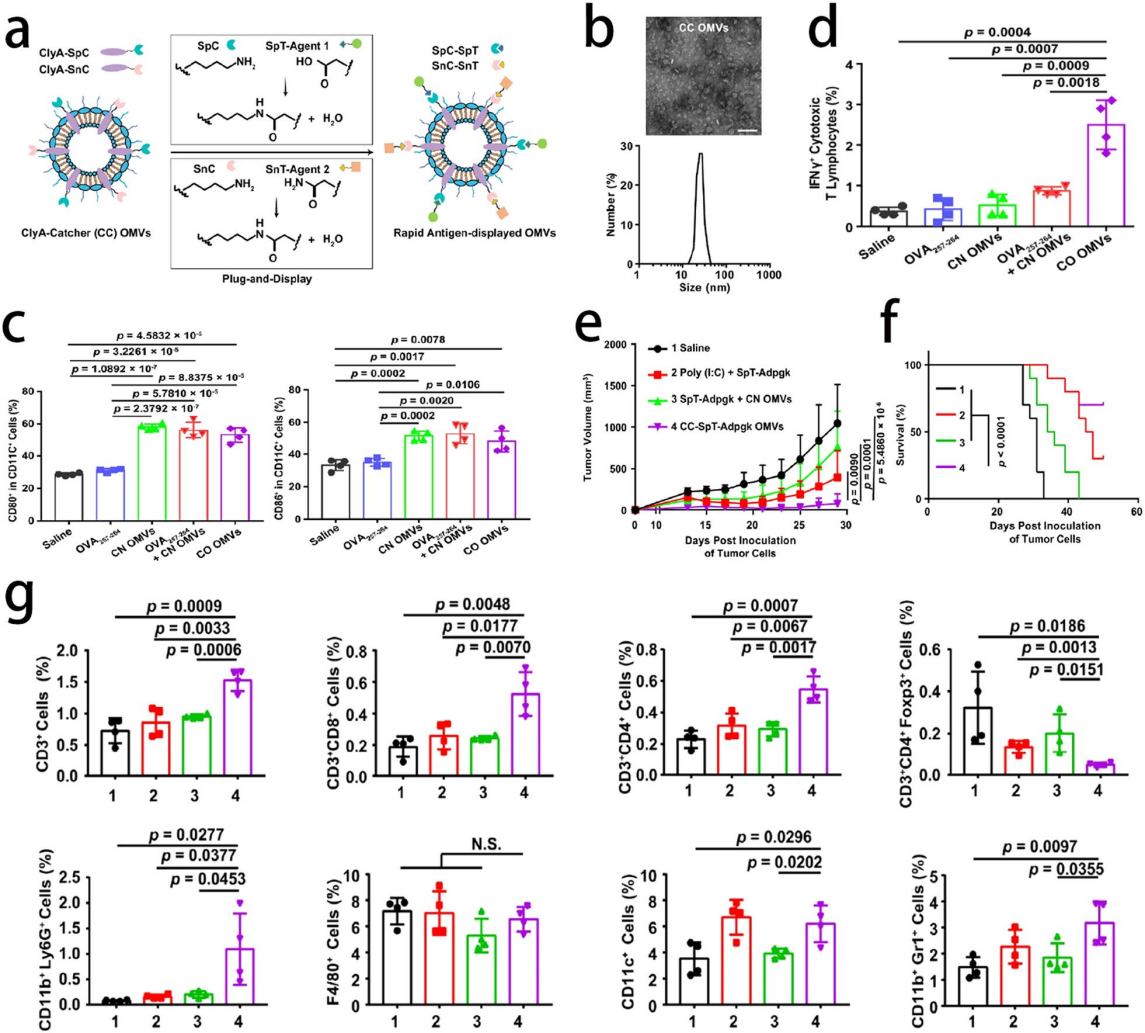
**a** Schematic illustration of ClyA-Catcher (CC) OMVs system for antigen display. **b** TEM and DLS analysis of CC OMVs. Scale bar, 100 nm. **c** The maturation status of DCs in inguinal lymph nodes on days 17 post immunization. The percentage of CD80^+^ and CD86^+^ cells in CD11c^+^ cells was assessed by flow cytometry. **d** Flow cytometry analysis of the percentage of IFN-γ^+^ cytotoxic T lymphocytes in splenocytes re-stimulated with OVA_257–264_ antigen. Tumor volumes (**e**) were recorded, and survival rate (**f**) was monitored after immunized with the indicated formulations on days 3, 7, and 11. **g** Tumors were harvested on day 29 for flow cytometry analysis (n = 4) of the following immune cells: CD3^+^, CD3^+^CD8^+^, CD3^+^CD4^+^, CD3^+^CD4^+^Foxp3^+^ T lymphocytes, activated neutrophils (CD11b^+^Ly6G^+^ cells), macrophages (F4/80^+^ cells), dendritic cells (CD11c^+^ cells), and MDSCs (CD11b^+^Gr1^+^ cells). The data are shown as mean ± SD. Statistical analysis was performed by two-tailed unpaired t test (e, g) and two-sided log-rank test (**f**). N.S. no significance. Reprint with permission from [98]. Copyright © 2021, Springer Nature

Keman et al. attempted to establish an OMVs-based flexible tumor vaccine platform to display target antigens by genetic engineering and “Plug-and-Display” technology [95–97]. They found that antigen can easily fuse with ClyA protein on the surface of OMVs. Therefore, this OMV-based tumor vaccine platform can stably integrate with antigens and efficiently accumulate in lymph nodes, which means that OMV-based tumor vaccines can efficiently deliver antigens to lymph nodes and present antigens to DCs, leading to antigen-specific T-lymphocyte-mediated anticancer immune responses [98] (Fig. 10). OMVs can break the tolerance of B cells and the surface of OMVs is rich in lipopolysaccharides (LPSs) and outer membrane proteins, which are the major components of pathogen-associated molecular patterns (PAMPs). This makes OMV a perfect natural adjuvant. Huang et al. modified basic fibroblast growth factor (BFGF), an angiogenic molecule, on the surface of OMVs. As an anticancer vaccine, BFGF-OMVs can break the tolerance of B cells, so that persistent and high levels of anti-BFGF autoantibodies can be produced by the immune system, which exhibits persistent, efficient, and multifunctional tumor suppression effects [99]. These studies showed that OMV, as a natural adjuvant, is one of the most suitable platforms for cancer vaccines.

Due to the existence of multiple biological barriers, the efficiency of nanodrug delivery will be greatly reduced. To overcome this issue, Li et al. reported an OMV-based nanoplatform that encapsulated NPs@Pt into OMVs. OMVs can be recognized and phagocytized by neutrophils, so that neutrophils can be a temporary carrier. According to the tendency of neutrophils to undergo inflammation, the author suggested that through photothermal therapy (PTT), an inflammatory region can be created in the tumor area, so that neutrophils can carry NPs@Pt into the tumor area. On the one hand, it can improve the delivery efficiency of drugs; on the other hand, it can make up for the incomplete curative effect of PTT [100].

Qing et al. found that OMVs would rapidly lead to serious systemic inflammatory responses via intravenous (i.v.) injection. To address this issue, the authors used calcium phosphate with high biocompatibility to encapsulate OMVs with pH sensitive nanoshells. In the slightly acidic microenvironment of the tumor, the calcium phosphate shell is dissolved, which can neutralize the acidic TME but can also expose OMVs to the tumor tissue and stimulate the local immune response [101].

To summarize, the biological characteristic of OMV makes it an ideal natural adjuvant, which will be beneficial for cancer immunotherapy. In addition, same as the other kinds of nanoparticles, OMV has the potential to load both hydrophilic and hydrophobic drugs, so that OMV as an adjuvant can combine other therapeutic methods (e.g., PDT, PTT, targeted therapy, etc.) to activate anticancer immunity. However, the drug encapsulation efficiency depends on the ability of drugs to cross the OMVs’ membrane barrier. What’s more, detoxification of OMV is an urgent problem to be solved for OMV-based nanoparticles and how to maintain a balance between detoxification and retaining enough efficacy of adjuvanticity is a topic that needs to be in-depth discussed [92].

### Extracellular vesicles in cancer immunotherapy

In 1980, Trams et al. first proposed the term “exosome” [102]. EVs include microvesicles, exosomes and apoptotic bodies. Studies have shown that almost all living cells can secrete EVs, which are isolated from various kinds of biological fluids [103, 104]. EVs express the same membrane proteins as the source cells, so tumor-derived extracellular vesicles (TEVs) have the potential to become tumor vaccines [105]. Muzaffer et al. used 4T1/Her2 cell-derived exosomes as a novel exosome-based therapeutic vaccine nanoplatform. To improve the immune response to TSAs on the surface of tumor derived exosomes (TEXs), the authors chose nucleic acid-based adjuvants to induce innate immunity. Consequently, CpG oligonucleotides (CpG ODNs) and polyinosinic-polycytidylic acid (p(I:C)) were co-encapsulated into 4T1/Her2 cell-derived exosomes, which could generate humoral and cell-mediated immune responses simultaneously [106]. A previous study showed that DCs and T cells can be significantly activated by single miRNAs (Let-7i, miR-155 and miR-142) [107], Adeleh et al. designed tumor-derived EVs encapsulated with multiple miRNAs (including Let-7i, miR-155 and miR-142), which could improve the survival rate and inhibit tumor growth [108].

**Fig. 11:**
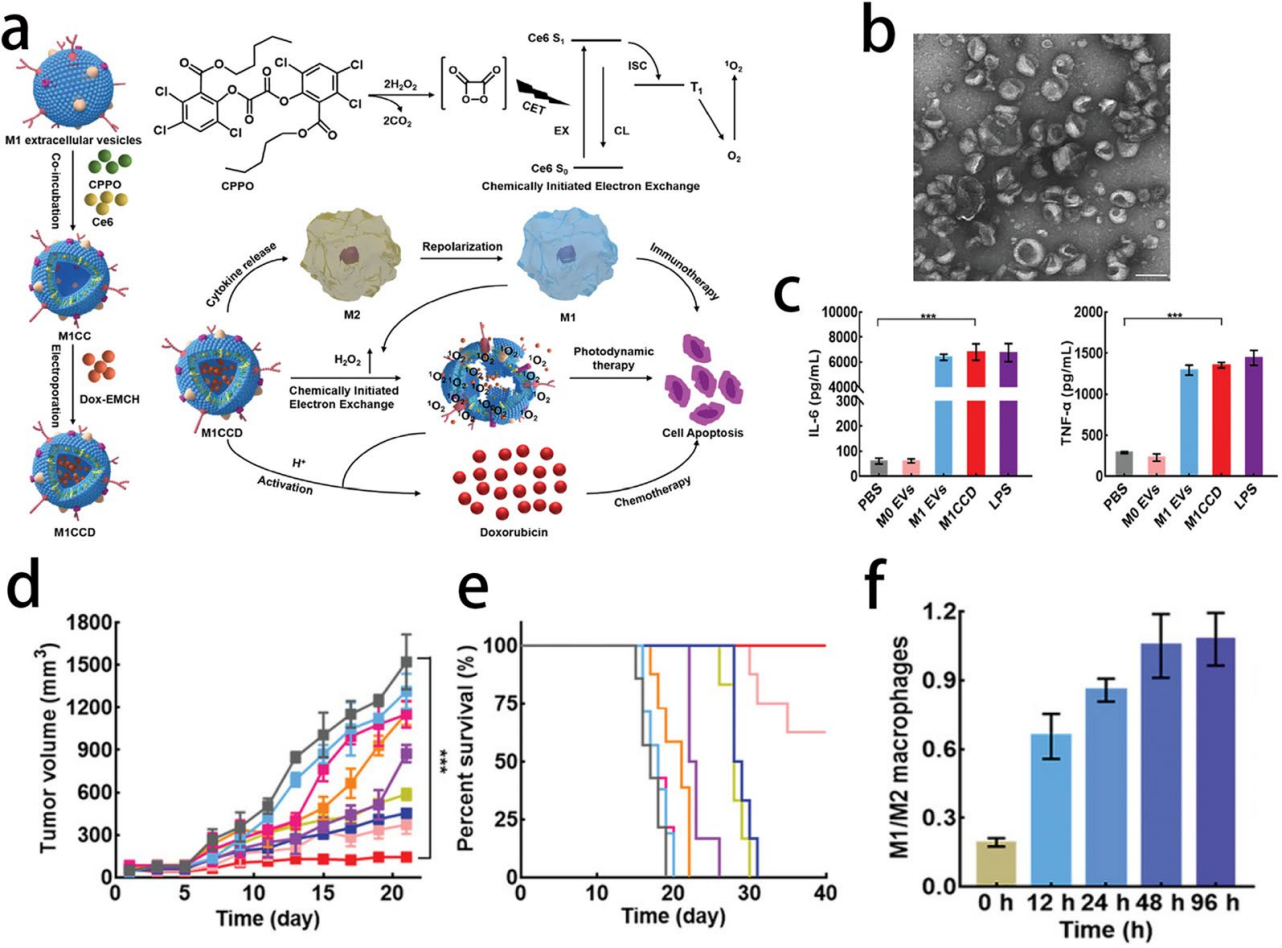
**a** Schematic illustration of the trimodal anticancer therapy by using M1CCD. **b** TEM image of M1CCD. Scale bar: 200 nm. **c** Production levels of IL-6 and TNF-α in M2 macrophages assayed by ELISA after different treatments for 24 h. **d** Tumor growth curves for all treated groups and (**e**) survival rates of tumor-bearing mice receiving different treatments in vivo. **f** Time-dependent ratios of the M1 phenotype to the M2 phenotype in tumor tissues after treatment with M1CCD. Reprint with permission from [114]

Immune cells-derived EVs, such as DCs, are also promising nanoplatforms for immunotherapy [109]. DC-derived small EVs, also named DC-sEVs, contain several immunologically relevant components, making DC-sEVs a novel candidate for cancer immunotherapy. However, DC-sEVs cannot induce sufficient anticancer immunity. To compensate make up for this defect, Akihiro et al. added ovalbumin (OVA) create DC-sEVs with high immunity, and added LPS and IFN-γ to prepare DC-sEVs with high immune activity. Then, sEVs were collected from activated DCs (also named activated-DC_OVA_-sEVs), and the activated-DC_OVA_-sEVs exhibited great therapeutic effects in tumor-bearing mice [110]. It is noteworthy that there are several basic researches and clinic trails reported that mature DC derived sEVs exhibited immunogenic potential and had capability to activate the T cells and NK cells. On the contrary, immature DC derived sEVs can induce Treg cells and even play an important role in maintaining peripheral tolerance [111–113]. Therefore, mature DC derived sEVs are more suitable as nanocarrier in cancer immunotherapy. Moreover, M1 macrophage-derived EVs (M1-EVs) also hold great potential in cancer immunotherapy. Ding et al. developed an M1-EV-based vehicle encapsulated with chlorin e6 (Ce6), prodrug aldoxorubicin (Dox-EMCH) and bis [2,4,5-trichloro-6-(pentyloxycarbonyl) phenyl] oxalate (CPPO), named M1CCD. After tail vein administration, M1-EVs can specifically target the tumor area and polarize macrophages from M2-like tumor-associated macrophages (M2-TAMs) to M1-like tumor-associated macrophages (M1-TAMs). In addition to the immune activation effect, H_2_O_2_ can also be produced. Chemical energy was generated by the reaction between H_2_O_2_ and CPPO, which can activate Ce6 without light and produce ROS. ROS can induce membrane rupture to release the prodrug Dox-EMCH, which can be activated by the acidic tumor microenvironment. The treatment strategy integrates immunotherapy, photodynamic therapy and chemotherapy, which enhances the therapeutic effect of cancer [114] (Fig. 11).

Additionally, adoptive T-cell therapy, such as chimeric antigen receptor T-cell (CAR-T) Immunotherapy, has emerged as a promising immunotherapy for various kinds of cancers [115]. Despite the unique therapeutic effect of CAR-T immunotherapy, it also has serious side effects that cannot be ignored, such as cytokine release syndrome (CRS) [116]. Fu et al. found that CAR-containing exosomes released from CAR-T cells express ample cytotoxic molecules and will not be weakened by anti-PD-L1 treatment. Moreover, CAR exosomes are much safer than CAR-T therapy in CRS models [117].

**Fig. 12:**
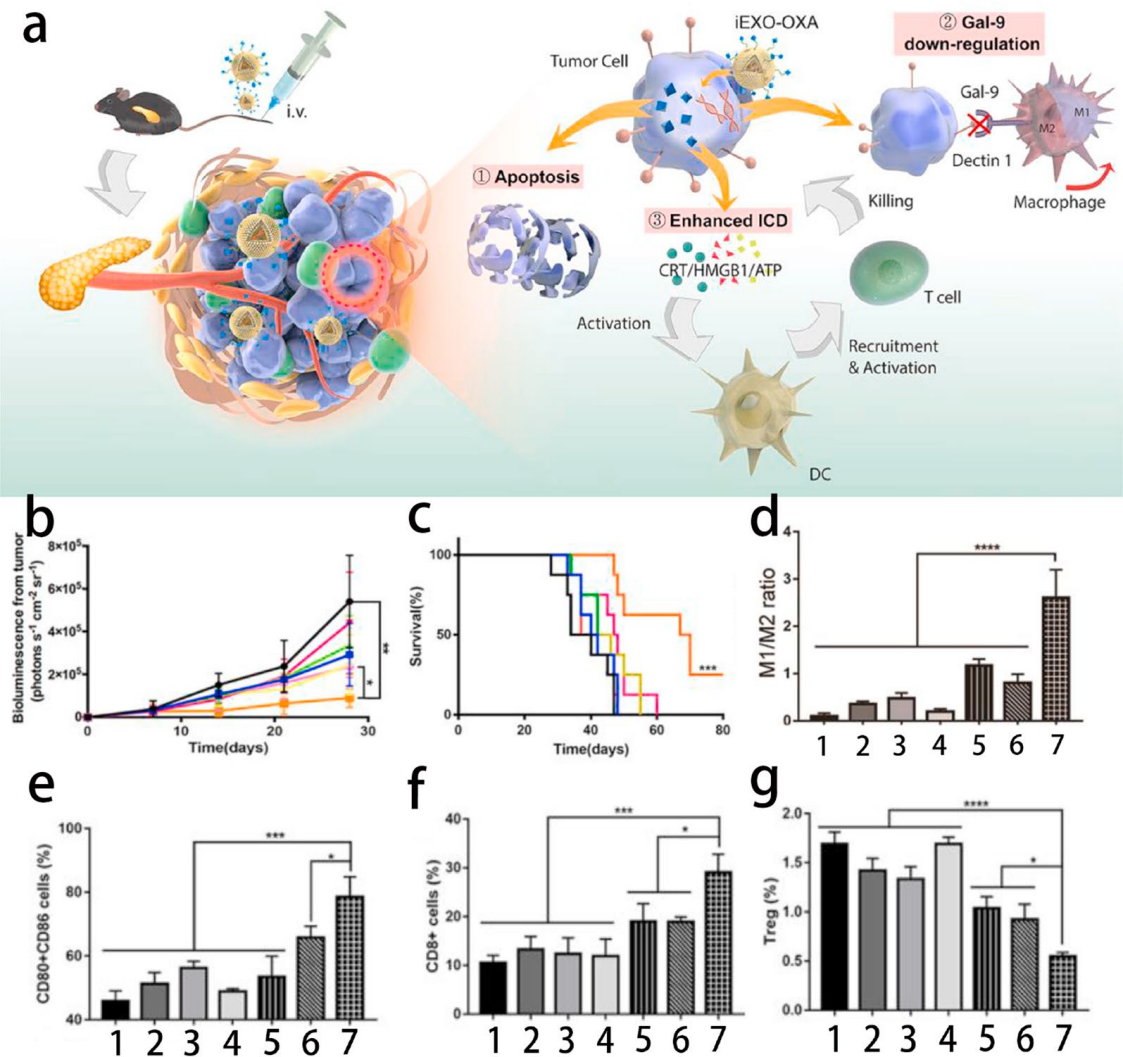
**a** Pancreatic cancer-targeting exosomes for enhancing immunotherapy and reprogramming tumor microenvironment. **b** The statistic of tumoral bioluminescence and **c** survival rate in vivo (n = 8, i.v., 5 mg OXA/kg, ~ 108 exosomes per mouse). **d** Statistical results of M1/M2 ratio (n = 3). Statistical results of (**e**) mature DCs, (**f**) CD8^+^T cells, and (**e**) Treg cells in orthotopic PANC-02 tumor-bearing mice after various treatments analyzed by flow cytometry. Data presented as means ± SD, one-way ANOVA, *p < 0.05, ***p < 0.001, ****p < 0.0001. (Groups: 1. PBS; 2. GEM; 3.OXA; 4. Scrbl-iEXO; 5. iEXO; 6. EXO-OXA; 7. iEXO-OXA). Reprint with permission from [120]. Copyright 2020, Elsevier Ltd

In recent years, it has been reported that bone marrow mesenchymal stem cells (BMSCs) or BMSC-derived exosomes have tumor-homing functions in several mouse models [118, 119], which makes BMSC and BMSC-derived exosomes promising nanovesicle drug delivery platforms. Zhou et al. developed a BMSCs-derived exosome-based nanovesicle, electroporation-loaded galectin-9 siRNA, for reversing tumor immunosuppression by M2-TAMs and modified it with ICD-triggered OXA-prodrug on the surface of exosomes (iEXO-OXA). This nanovesicle drug delivery system can elicit anticancer immunity by reversing M2-TAMs polarization, cytotoxic T lymphocyte recruitment and Treg downregulation and exhibits excellent therapeutic effects [120] (Fig. 12).

In conclusion, EVs inherit the biological characteristics of the originated cells. Among all kinds of EVs, immune cells-derived EVs play a significant role in tumor immune microenvironment and can be used as promising candidate nanocarriers for cancer immunotherapy. Notably, that the immature DCs and M2 macrophages derived EVs would promote the tumor suppressive immune microenvironment, so they are not recommended as nanocarriers in tumor immunotherapy. Besides, although tumor-derived EVs can stimulate the anticancer immunity, tumor-derived EVs are still considered to be involved in tumorigenesis and immune escape. Similar to tumor-derived EVs, BMSC-derived EVs show good biosafety, but BMSC-derived EVs would reduce DCs maturation, promote the polarization of M2 macrophages and increase the infiltration of Tregs. So, if we attempt to use tumor-derived EVs or BMSC-derived EVs as the nanocarriers in cancer immunotherapy, we need to weigh the pros and cons [121]. Moreover, the drug encapsulation efficiency of EVs is limited, and also has the disadvantages of low yield and high cost, which hinders the progress of clinical translation.

### Hybrid nanovesicles in cancer immunotherapy

**Fig. 13:**
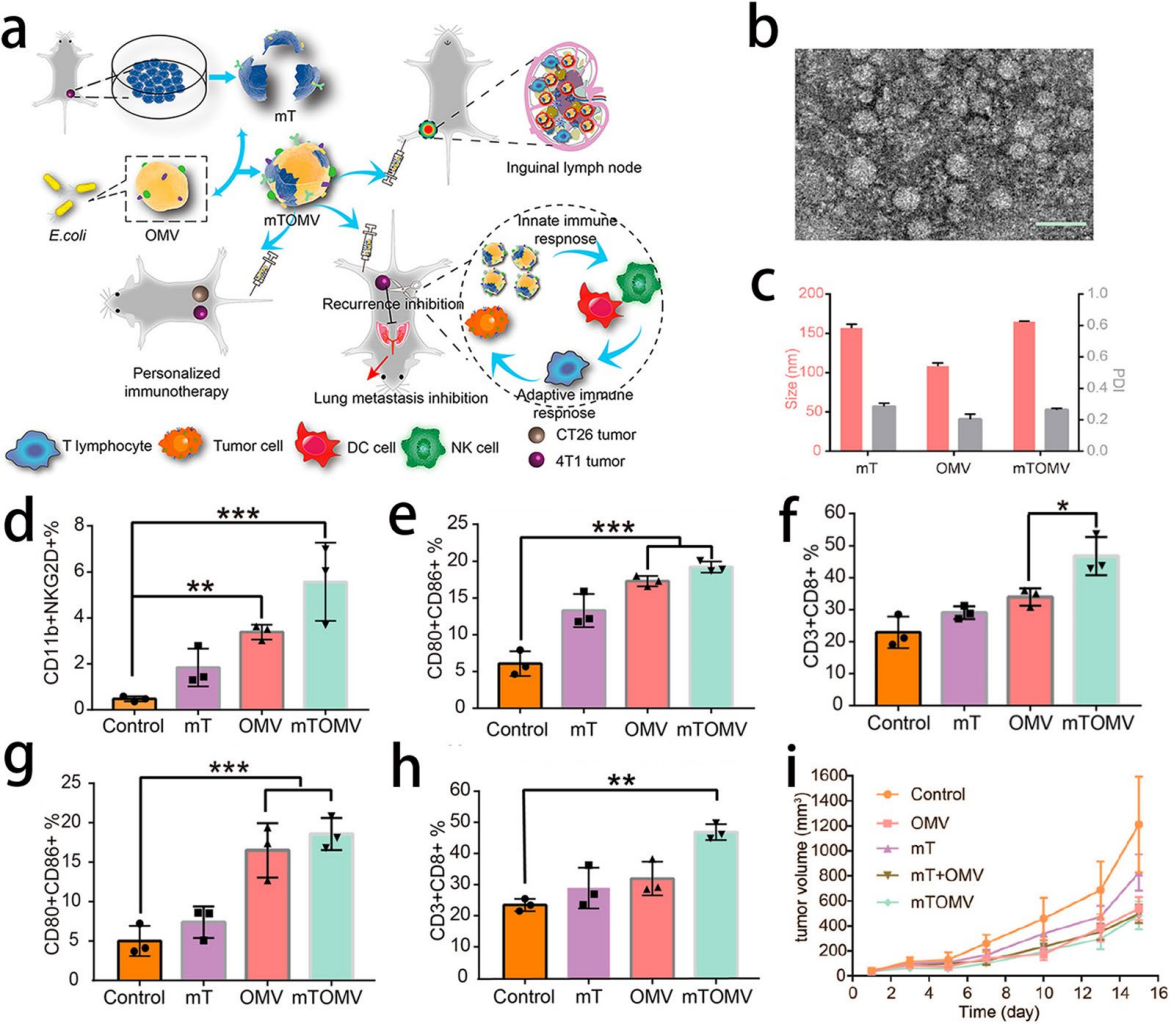
**a** Schematic Illustration of the Hybrid Vesicles from Bacteria Outer Membrane and Tumor Cell Membrane to Enhance Innate Immune Response for Personalized Immunotherapy. **b** TEM image of mTOMV. Scale bar = 50 nm. **c** Hydrodynamic size distribution and PDI of mT, OMV, and mTOMV. **d** The percentage of CD11b^+^NKG2D^+^ cells in 4T1 tumors (n = 3). **e** The percentage of CD80^+^CD86^+^ cells in 4T1 tumors (n = 3). **f** The percentage of CD3^+^CD8^+^ cells in 4T1 tumors (n = 3). **g** The percentage of CD80^+^CD86^+^ cells in lymph nodes (n = 3). **h** The percentage of CD3^+^CD8^+^ cells in lymph nodes (n = 3). **i** The relative tumor volume of CT26 tumors treated with control, mT, OMV, mT + OMV, and mTOMV, respectively (n = 7). Data are represented as mean ± SD. Statistical significances were calculated via one-way ANOVA, *p < 0.05, ***p < 0.001, ****p < 0.0001. Reprint with permission from [126]. Copyright 2021, Wiley‐VCH GmbH

The nanovesicles mentioned above have their own characteristics. Fusion of different types of nanovesicles can compensate for the defects of single nanovesicle [122]. Among them, bacterial OMVs have strong immunogenicity and natural adjuvant properties, which can play an important role in tumor immunotherapy. To improve the efficacy of other types of nanovesicles in cancer immunotherapy, bacterial OMVs are often mixed with other types of nanovesicles to form hybrid vesicles to participate in cancer immunotherapy. Zhai et al. designed a novel biomimetic nanoplatform, named PLOVs, by fusing OMVs and photosensitive liposomes carrying CD38 siRNA (PTSLs). PTSLs can induce ICD by PTT and enhance the function of T cells by CD38 siRNA. Moreover, OMVs can enhance the immune response through their own adjuvant effect [123]. Wang’s group and Chen’s group both developed OMV-cancer cell member vesicle (OMV-CMV) nanoplatforms with PTT. CMVs have homing characteristics and can specifically target tumors, and the TSA on the surface of CMVs can stimulate the anticancer immunity through mature APCs. However, due to the inability to effectively stimulate the maturation of APCs in the TME, it is necessary to use immune stimulants to stimulate the maturation of APCs. Fortunately, as a natural adjuvant, OMVs can accomplish this function. Both OMV-CMVs exhibited excellent targeting ability and immune activation ability and achieved good cancer immunotherapy effects [124, 125]. Similar to the strategies mentioned above, Zou et al. designed a personalized immunotherapy strategy and formed a new functional vesicle (mTOMV) by hybridizing an OMV with the cell membrane from a tumor (mT). mTOMV effectively inhibits the growth and metastasis of tumors with a simple preparation procedure and good biocompatibility [126] (Fig. 13).

**Fig. 14:**
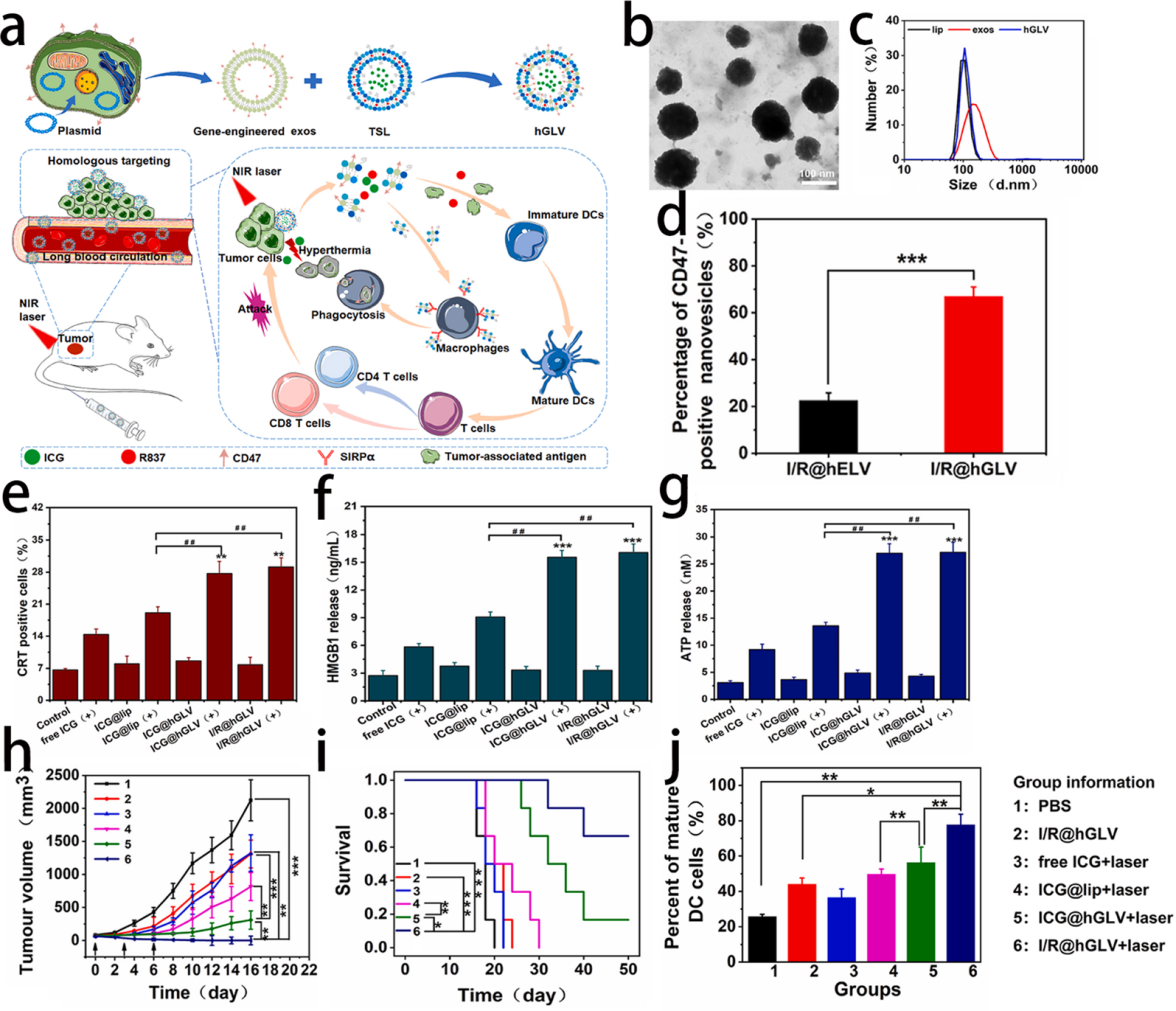
**a** The design principle of hGLV and the antitumor mechanism of hGLV through PTT combined with immunotherapy. **b** A typical morphology of hGLV obtained by TEM. **c** Size distribution of lip, exos and hGLV. **d** Quantitative of the expression of CD47 on the surface of I/R@hELV and I/R@hGLV by Flow Nano Analyzer. ICD evaluation. **e** Flow cytometric analysis of CRT exposure on the surface of CT26 cells, **f** The levels of HMGB1 and **g** ATP levels in the supernatant of CT26 cells. Antitumor Efficacy of I/R@hGLV plus laser in vivo. **h** Tumor volume growth curves and (**i**) Kaplan–Meier survival analysis of the mice bearing CT26 tumors after treatment. Quantitative analysis of DCs maturation in the spleens induced by different treatments on mice bearing CT26 tumors. ***p < 0.001, **p < 0.01, *p < 0.05. Reprint with permission from [129]. Copyright 2021, Elsevier Ltd

As the most successful nanovesicles in clinical translation, liposomes possess good biosafety and drug loading efficiency. Natural nanovesicles, such as exosomes can specifically express some proteins by genetic engineering of source cells, but with insufficient encapsulation of drugs [127, 128]. Cheng et al. designed hybrid therapeutic nanovesicles, which fused gene-engineered CD47 overexpressing exosomes with drug-loaded thermosensitive liposomes, named hGLV. By fusing liposomes with exosomes, the difficulty of liposome-modified proteins and the problem of the low drug loading rate of exosomes were solved. The novel hybrid nanovesicle exhibited the long blood circulation and enhanced the phagocytosis of cancer cells by macrophages, achieved a great photothermal treatment effect and effectively inhibited the growth of tumors [129] (Fig. 14).

In brief, hybrid vesicles can combine the advantages of different types of vesicles to achieve a better therapeutic effect than single type of vesicles do. However, it still faces the disadvantages of high production cost and low yield. Moreover, it is more difficult to prepare hybrid vesicles than single type of vesicles, which makes the clinical translation of hybrid vesicles much harder than the other type of vesicles we mentioned before.

## Concluding and future perspectives

As one type of lipid-nanovesicle (LNV), the clinical application of liposome chemotherapy drugs has greatly improved the survival time of cancer patients. Because they have the same lipid bilayer structure as cells, LNVs possess great biocompatibility, which means that LNVs have great potential for translation. In addition, immunotherapy has been a great success in cancer therapy, but the immunotherapeutic drugs approved by the FDA are not effective for all kinds of cancer, and only a minority of patients benefit from it. Moreover, some immune checkpoint blockade (ICB) treatments can cause serious systemic side effects [130, 131]. This makes us wonder whether LNDDSs can be used for cancer immunotherapy to solve the current problems of immunotherapy. For this reason, we summarize the latest advances in LNDDSs in cancer immunotherapy.

Liposomes, as the star product of LNVs, possess good biocompatibility. In addition, compared with other kinds of LNVs, liposomes have better drug loading capacity regardless of whether the drug is hydrophobic or hydrophilic [132]. Moreover, in order to obtain the biological function of liposomes and achieve the purpose of cancer immunotherapy or targeting tumors, we need to modify the surface of liposome membrane with proteins or peptides through postinsertion or chemical bonds. Although liposomes have a complete and mature preparation process, the complex modification process and difficult purification still bring challenges to the clinical translation of engineered liposomes, which will limit the application of liposomes in cancer treatment.

Bacterial outer membrane vesicles are derived from gram-negative bacteria, so that OMVs can stimulate a nonspecific immune response, which makes OMVs a natural adjuvant. According to existing studies, OMVs are most suitable as a perfect auxiliary component of tumor vaccines. Due to their adjuvant property, OMVs need to combine with other anti-cancer treatments to activate specific anti-cancer immunity. Also, it’s more suitable for OMVs to combine with other nanovesicles to form hybrid vesicles for cancer immunotherapy. OMVs also have an obvious disadvantage: the endotoxins of OMVs will lead to a systemic immune response, so they need to be attenuated, while retaining sufficient adjuvant effect. To address this issue, it is necessary to quantify the toxicity reduction of OMVs in the future to ensure their toxicity is in a controllable range while maintaining its adjuvant effect.

Cell membrane vesicles and EVs also have great clinical translation potential. Among the different kinds of cell-derived vesicles, tumor-derived cell membrane vesicles and tumor-derived EVs both express TSAs on the surface of membranes and can be used as tumor vaccines for cancer immunotherapy. However, although TSAs are located on the surface of vesicle membranes, it is still necessary to stimulate the tumor microenvironment and activation of immune cells (e.g., APCs, T cells, macrophages) to achieve the ideal therapeutic effect. In addition, PD-L1 expressed on the surface of tumor cell membrane is a recognized mechanism of tumor immune escape which may lead to tumor progression and even the emergence of local metastasis. In particular, Chen et al. found that tumor exosomal PD-L1 would suppress the function of CD8 T cells extremely hinder the development and application of this kind of LNV [133]. Moreover, except for some immunosuppressive cell-derived cell membranes and immunosuppressive cell-derived EVs, such as immature DCs-derived vesicles, M2 macrophages-derived vesicles and so on, most immune cell-derived cell membrane vesicles and immune cell-derived EVs can induce anticancer immunity, but the anticancer effect is very limited. Therefore, this therapeutic strategy needs to be combined with other therapeutic methods to produce a stronger immune response in cancer. Conventional cell-derived vesicles include BMSC-derived vesicles, platelet-derived vesicles, H293T cell-derived vesicles, etc. Compared with other cell-derived vesicles mentioned above, conventional cell-derived vesicles can’t directly stimulate cancer immunity. In particular, BMSC-derived vesicles will induce tumor suppressive immune microenvironment. However, conventional cell-derived vesicles also possess the advantages other cell-derived vesicles don’t have. For example, BMSC-derived vesicles have homing ability and can target tumor sites specifically, platelet-derived vesicles can accumulate in surgical wound sites and target CTCs in the blood, which is suitable for the patients after tumor surgery, H293T cell-derived vesicles can be engineered by transfecting the H293T cell, which is easy to transfect and also known as the “tool cell”. What’s more, it is worth noting that most cell membrane vesicles are originated from cell lines, which may induce immune rejection in model mice or patients. Additionally, high cost and low yield also restrict the clinical translation of cell membrane vesicles and EVs.

In summary, we introduced LNVs and the application progress in cancer immunotherapy. LNVs, as nanoparticles with clinical translation potential will benefit patients by large-scale application in the future. The biggest issue facing liposomes is that compare with the other LNVs it is complex to modify the proteins on their surface, and it is difficult to purify after modification. OMVs and tumor-derived vesicles are candidates for tumor vaccines. However, OMVs need to find a balance between reducing toxicity and maintaining adjuvant function. Tumor derived vesicles can promote the occurrence and development of tumors and may lead to tumor metastasis. Researchers need to weigh the benefits of tumor derived vesicles-based cancer immunotherapy for patients. For most EVs and OMVs, there are many methods to encapsulate drugs, such as electroporation, co-incubation, sonication and so on, however, the low drug encapsulation efficiency is an urgent problem needs to be solved. For the LNVs mentioned in this review, it is limited in clinical translation owing to its high cost, low yield and huge workload. Once we overcome this problem, EVs will have broad application prospects. In the future, novel and multifunctional nanoplatforms based on LNVs will be developed, and we are confident in LNVs’ clinical translation for cancer immunotherapy.

## Abbreviations

LNDDS: Lipid nanovesicle drug delivery system
PDT: Photodynamic therapy
EPR: Enhanced permeability and retention
FDA: Food and drug administration
OMV: Outer membrane vesicle
PDAC: Pancreatic ductal adenocarcinoma
EV: Extracellular vesicle
mAb: Monoclonal antibody
CAR: Chimeric antigen receptor
PD-1: Programmed death-1
PD-L1: Programmed death ligand 1 ()
EpCAM: Epithelial cell adhesion molecule
IFN-γ: Interferon-γ
IL-6: Interleukin-6
NK: Nature killer
DC: Dendritic cell
TAM: Tumor-associated macrophage
Treg: T regulatory cell
MDSC: Myeloid-derived suppressor cell
MR: Mannose receptor
TIME: Tumor immune microenvironment
ICD: Immunogenic cell death
CHI: Chidamide
MHC-I: Major histocompatibility complex-I
TNBC: Triple-negative breast cancer
ROS: Reactive oxygen species
Met: Metformin
DOX: Doxorubicin
IDO-1: 2,3-Dioxygenase 1
IND: Indoximod
MTO: Mitoxantrone
COVID-19: Coronavirus disease-19
mRNA: Messenger ribonucleic acid ()
TMV: Tumor membrane vesicle
CTLA-4: Cytotoxic T-lymphocyte-associated protein 4
DNA: Deoxyribonucleic acid
NV: Nanovaccine
TOPSi: Thermally oxidized porous silicon
AcDEX: Acetalated dextran
SpAcDEX: Spermine-modified AcDEX
CP: Cyclophosphamide
LPS: Lipopolysaccharide
PAMPs: Pathogen-associated molecular patterns
BFGF: Basic fibroblast growth factor
PTT: Photothermal therapy
TEV: Tumor-derived extracellular vesicle
TEX: Tumor derived exosome
CpG ODN: CpG oligonucleotide
p(I:C): Polyinosinic-polycytidylic acid
CPPO: Bis [2,4,5-trichloro-6-(pentyloxycarbonyl) phenyl] oxalate
CAR-T: Chimeric antigen receptor T-cell
CRS: Cytokine release syndrome
BMSC: Bone marrow mesenchymal stem cell
CMV: Cancer cell member vesicle
APC: Antigen-presenting cell
LNV: Lipid-nanovesicle
ICB: Immune checkpoint blockade
TSA: Tumor special antigen
TAAs: Tumor associated antigens
